# Promoter antisense RNAs: beyond transcription by-products of active promoters

**DOI:** 10.1080/15476286.2022.2062177

**Published:** 2022-04-15

**Authors:** Fan Yang

**Affiliations:** Department of Medicine, University of California, San Diego, La Jolla, California, USA

**Keywords:** Promoter antisense RNA, Pol II promoter-proximal pause release, nascent RNA sequencing, CRISPR-Cas9, CRISPR-Cas13

## Abstract

Omics-based technologies, which have developed rapidly over the last few decades, have generated increasing evidence demonstrating pervasive divergent transcription from RNA polymerase II (Pol II) promoters of eukaryotic genome, and indeed have raised considerable discussion as to their potential physiopathological function. Unlike many other long non-coding RNAs (lncRNAs), promoter antisense RNAs (PAS RNAs) were initially considered to be merely passive transcription by-products of active promoters. However, recent studies have begun to reveal their critical importance in a broad spectrum of biological processes. In this Review, I summarize recent technological advances that enable accurate detection of PAS RNA and discuss the mechanisms of PAS RNA biogenesis emphasizing the functional importance of its structure enabling the diverse functions of PAS RNA in transcription and chromatin regulation.

## Introduction

**Figure 1.**
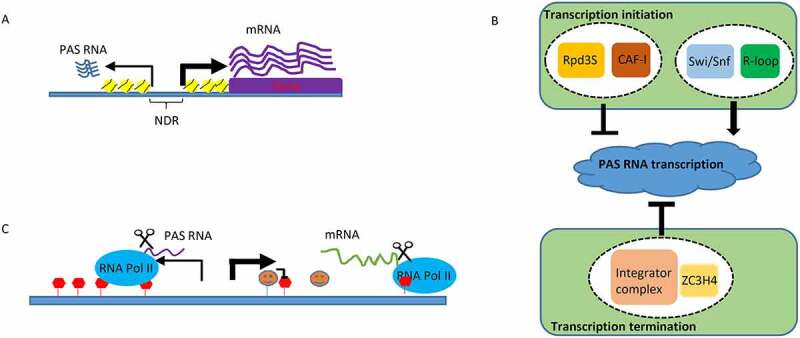
PAS RNA and its regulation

Demonstration of a massive lncRNA transcriptional program in the mammalian genome has conceptually challenged our understanding of central dogma in molecular biology and has sparked heated debate about their biological functions [1–4]. While the function of many lncRNAs, as well as miRNAs, piRNAs and many other types of non-coding transcripts is supported by extensive evidence [5–7], there still remains considerable debate about the potential functions of PAS RNAs [8–10], a class of non-coding antisense transcripts that originate from shared bidirectional promoters (Figure. 1(a)). Indeed, bidirectional transcription from active Pol II is widespread in a large number of eukaryotic organisms [11–13]. A great proportion of human and murine lncRNAs found in embryonic stem cells and other cell types originate from divergent transcription of active Pol II promoters [9,14]. Distinct from messenger RNAs (mRNAs), PAS RNAs are generally expressed at low levels, unstable, and lack protein coding potential, which raises an early hypothesis that pervasive expression of PAS RNAs reflects consequence of active promoters and merely transcription noise [15]. However, accumulating evidence demonstrates that PAS RNAs, which are coordinately expressed with paired mRNAs upon differentiation as reported, are intrinsically and dynamically regulated [9,16,17]. These findings raise another intriguing question: Is there any potential functional importance of the non-coding antisense transcript at the promoter of many or most coding transcription units, and if so, what is the role this might play in regulated promoter activation events?

In this Review, I begin with a comparison of the two most powerful methods to detect PAS RNA, and discuss the inherent advantages and disadvantages of each method followed by a review of how PAS RNA is regulated. I also discuss the mechanisms of PAS RNA-mediated gene regulation, primarily focusing on the PAS RNA secondary structure–mediated transcription activation. Furthermore, I propose the potential mechanism(s) that PAS RNA might employ to execute its biological function and emphasize the key challenges for studying the pathophysiological functions of PAS RNA.

## High-throughput methods to detect PAS RNA

Almost all the conventional genome-wide transcriptomic techniques only detect stable transcripts in the steady state by purification and enrichment of polyadenylation (poly[A]) tail-containing RNA. Most of PAS RNAs are difficult to identify with conventional RNA sequencing (RNA-seq) approach due to the intrinsic degradation of nuclear RNA exosome complex (details described in the next section: regulation of PAS RNA) [18]. Reverse transcription followed by quantitative polymerase chain reaction (RT-qPCR) and RNA fluorescence in situ hybridization (FISH) are capable of detecting expressed PAS RNAs successfully. However, both methods only allow the target analysis of PAS RNAs and are limited by low throughput. Ascribed to the technological development of high-throughput sequencing based techniques capable of investigating nascent RNA synthesis, the advent of nascent transcripts sequencing methodologies has unveiled pervasive transcription in the mammalian genome, including transcripts that arise from divergent transcription of active promoter regions [19–22]. In general, these nascent RNA sequencing methods require the following steps to achieve genome-wide identification and characterization of newly transcribed RNAs: (1) isolation of nascent transcripts; (2) reverse transcription; (3) library preparation and sequencing; (4) quality control of sequencing data; and (5) mapping reads to reference genome for quantification. Based on the different means for isolating nascent transcripts, these methods can be generally classified into two groups: (a) to isolate nascent transcripts via biochemical enrichment; (b) to probe RNA synthesis via biochemically inducing nucleotide point mutations. Despite shared library preparation principle among different approaches, distinct nascent RNA sequencing methods vary remarkably in their ability to enrich and detect PAS RNAs. Herein, I summarize two of the most powerful methods in the detection of PAS RNAs by pinpointing the sensitivity, resolution and shortcoming of each method. For a clearer understanding of nascent RNA based sequencing technologies, I refer readers to other outstanding reviews that critically evaluate distinct strategies used for measuring nascent transcription [23–25].

### Run-on RNA sequencing

In 2008, Leighton J. Core *et al*., developed global run-on sequencing (GRO-seq) method to specifically enrich genome-wide nascent RNAs and unravelled a plethora of PAS RNAs from primary human lung fibroblast IMR90 cells [21]. This run-on experiment begins with cell nuclei isolation to avoid endogenous nucleotides, and proceeds to *in vitro* nuclear run-on reaction that marks nascent RNAs with 5-bromouridine 5’-triphosphate (brUTP), followed by specific purification and isolation of nascent RNAs using an antibody against brUTP for deep sequencing. Taking advantage of a well formulated run-on assay *in vitro*, anionic detergent sarkosyl and brUTP ensures that only nascent RNAs from transcriptionally competent Pol II are detected. Moreover, the *in vitro* run-on experiment is limited to 5 minutes, enabling efficient detection of rapid and dynamic Pol II transcriptional activity, and exceptionally robust measurement of highly labile lncRNAs, including PAS RNAs [26]. Notably, an updated derivative of GRO-seq, precision run-on sequencing (PRO-seq) approach in which biotin labelled nucleoside 5′-triphosphates (biotin-11-NTPs) are used to enrich newly synthesized RNAs in the run-on reaction step, provides a direct measurement of nascent RNAs at single nucleotide resolution [20]. Therefore, PRO-seq is a more powerful and sensitive genome-wide technique to robustly measure unstable nascent transcriptome, including PAS RNAs, permitting further mechanistic studies of related transcriptional regulation. Although powerful, as with all technologies, run-on methods have their own limitations. A major concern with the *in vitro* run-on reaction is that the observed transcriptional profiles from run-on experiments might not represent *in vivo* transcriptional activity and status of RNA polymerases. In addition, a large amount of starting material is required, and the experimental protocol is relatively laborious. Despite the fact that genome-wide run-on RNA sequencing approaches can robustly and precisely map the nucleotide position and regulation of PAS RNAs, they do not yet detect cell-to-cell heterogeneity across the cell population or provide spatial expression pattern of PAS RNAs within the nucleus.

### Transient transcriptome sequencing (TT-seq)

Instead of using brUTP or biotin-11-NTPs to enrich nascent transcripts in run-on RNA sequence approaches, TT-seq method utilizes membrane-permeable ribonucleotide analog, such as 4-thiouridine (4sU), to incorporate into newly transcribed RNA molecules by active RNA polymerases in 5 minutes labelling reaction [22]. As RNA fragmentation is conducted before affinity purification of 4sU labelled RNAs, TT-seq approach only identifies newly synthesized 3’ end RNA fragments during the period of 4sU labelling. By application of TT-seq to human K562 cells, Schwalb, B. *et al*., detected hundreds of PAS RNAs with distinct RNA synthesis rate and RNA half-lives [22]. Compared to run-on methods, TT-seq technique requires less starting material. Importantly, recent development and innovation of TT-seq technologies are particularly exciting as they have been shown to work not only in living cells, but also in living model organisms, including *Arabidopsis thaliana*, nematodes, zebrafish and even mice [27–30]. This is of considerable significance for the application of PAS RNAs in different biological systems [31]. In depth analysis in the functionality of PAS RNAs via various *in vivo* organismal models will undoubtedly provide novel mechanistic insights of PAS RNA- mediated gene regulation. However, TT-seq approach shows lower sensitivity to detect PAS RNAs than run-on sequencing approaches do. Although TT-seq restricts metabolic RNA labelling time to 5 minutes, PAS RNAs whose half-lives are less than 5 minutes might undergo rapid degradation before being captured by affinity purification and thus are too low to be robustly detected by TT-seq method. Like run-on methods, TT-seq based approaches do not provide spatial map information of PAS RNA expression in the three-dimensional space of the nucleus.

Overall, both methods just described above are capable of genome-wide detection and quantitation analysis of PAS RNA expression, offering versatile information into the mechanistic insights of PAS RNA regulation at specific stages of gene regulation. Each method, however, comes up with its own strengths and weaknesses. Run-on sequencing approaches are more sensitive in terms of their ability to detect PAS RNAs at a genome-wide level, allowing systematic investigation of PAS RNA regulation at different steps of transcription. TT-seq based methods, on the other hand, outperform run-on sequencing approaches due to the successful application of PAS RNA measurement *in vivo*, albeit at the expense of detection sensitivity. Furthermore, unlike run-on sequencing approaches in their ability to investigate PAS RNA expression in transcriptional regulation, TT-seq method and its derivatives are capable of assessing the half-lives of PAS RNAs. It should be noted that neither run-on sequencing nor TT-seq method is capable of quantifying PAS RNA expression in the three-dimensional space of the nucleus or monitoring the transcription dynamics of PAS RNA at the single-cell level. With the recent development of image based methods that allow hundreds of thousands of RNA molecules to be simultaneously detected and spatially resolved at single-cell transcriptomic level, I anticipate that Multiplexed Error-Robust Fluorescence In Situ Hybridization (MERFISH) [32] or RNA sequential Fluorescence In Situ Hybridization (seqFISH) [33], in combination with current genome-wide nascent RNA sequencing approaches, will provide a more sensitive and integrated view of PAS RNA expression landscape in both cell cultures and organisms as well as broaden our understanding of PAS RNA transcriptional and posttranscriptional regulation.

## Regulation of PAS RNA

As a key source of nuclear lncRNAs, unravelling how PAS RNA is regulated is of great importance. Distinct from their cognate protein-coding mRNAs, whose mature transcripts are mainly located in the cytoplasm, PAS RNAs are generally found in the nucleus and are closely associated with the transcribed chromatin regions. It has been well established that PAS RNA expression, as with mRNA expression, is intricately regulated via both transcriptional and post-transcriptional regulation. However, due to their unique localization and expression pattern, distinct machineries are involved in the precise regulation of PAS RNA expression in a variety of contexts. For example, although transcription initiation of most of PAS RNAs typically takes place at the same promoter region with their cognate coding gene transcripts, two separate transcription pre-initiation complexes (PICs) are assembled for transcription of PAS RNA and its cognate coding gene transcript, respectively [34]. This implicates that distinct transcription initiation mechanisms are employed for PAS RNA regulation. Indeed, it has been reported that the Rpd3S deacetylation complex, which is a histone H4 deacetylation complex that links Rpd3S-mediated deacetylation to PAS RNA transcription initiation regulation, strongly suppresses transcription in the antisense direction of active promoters [35]. In particular, chromatin assembly is also a critical regulator of PAS RNA transcription initiation [16]. In this chromatin-based model, PAS RNA transcription is modulated by the relative rates of both nucleosome assembly and disassembly at the −1 nucleosome at the promoter site. Specifically, the nucleosome assembly complex chromatin assembly factor 1 (CAF-I), facilitates incorporation of H3K56ac (histone H3 acetylated at lysine 56) modified nucleosome at promoter, and thus ensures potent repression of PAS RNA expression. Failure to deacetylate H3K56ac at the −1 nucleosome stimulates nucleosome eviction which is remodelled by chromatin remodelling complex Swi/Snf (SWItch/Sucrose Non-Fermentable). Therefore, loss of CAF-I decreases nucleosome occupancy at −1 position of promoter, leading to robust divergent transcription. On the contrary, loss of Swi/Snf attenuates eviction of −1 nucleosome and thus decreases PAS RNA transcription. Intriguingly, R-loops, three stranded nucleic acid structures formed when nascent transcript invades DNA duplex to form RNA:DNA hybrid and then displaces the coding strand as single stranded DNA (ssDNA), act as intrinsic Pol II promoters to promote antisense transcription [36]. In this study, Tan-Wong et al., initially, designed an elegant *in vitro* cell free experiment to demonstrate that a made R-loop in a plasmid was able to initiate transcription within the R-loop region. The authors then provided *in vivo* evidence to show that a large number of PAS RNAs co-localized with R-loop regions and that a great proportion of PAS RNAs mapping to R-loop regions exhibited decreased expression upon removal of R-loops by RNase H1 expression and increased expression upon induction of R-loops. Finally, the authors performed a preliminary mechanistic analysis to demonstrate that R-loops could promote recruitment of PICs specifically for PAS RNA transcription initiation, but not for sense coding transcripts transcription initiation. In addition to transcription initiation regulation, our understanding of how PAS RNA is regulated via transcription elongation and termination lags far behind. While it would be interesting to determine whether and how transcription elongation regulation affects PAS RNA transcription, several studies have reported that transcription termination regulation acts as another means to modulate PAS RNA expression. For example, the integrator complex, a complex that controls termination of many non-coding transcripts, such as small nuclear RNAs (snRNAs) [37], is also involved in termination of at least a subset of PAS RNAs [38] and enhancer-derived RNAs (eRNAs) [39]. In a proteomic screen aimed at identifying new transcription termination factors, ZC3H4, a metazoan zinc finger containing protein, was reported to facilitate early transcriptional termination of a cohort of non-coding transcripts, including PAS RNAs [40]. These studies provide additional evidence for the complexity of PAS RNA transcriptional regulation and, indeed, has deepened our understanding of PAS RNA formation. Taken together, these studies reveal versatile transcription regulatory mechanisms for cells to control PAS RNA homoeostasis (summarized in Figure. 1(b)).

In addition to transcriptional regulation, another well-known mechanism for controlling PAS RNA expression is through RNA degradation driven by the nuclear RNA exosome complex [18]. The eukaryotic nuclear exosome is a multifactorial 3’-5’ exoribonucleolytic complex that plays an important role in a variety of critical biological processes, such as surveillance of versatility and specificity of a large cohort of RNA substrates [41]. Among them, a substantial fraction of unstable lncRNAs, PAS RNAs, are monitored by the nuclear RNA exosome complex, and their decay is closely regulated by the nuclear RNA exosome-mediated rapid RNA degradation. The fact that the nuclear RNA exosome complex depletion leads to widespread expression of PAS RNAs raises an intriguing question: how are low abundant PAS RNAs specifically recognized and targeted by this complex? This is partially explained by the important finding that promoter directionality is determined by asymmetric distribution of poly(A) sites and U1 small nuclear ribonucleoprotein (snRNP) recognition sites. The high density of poly(A) sites in the upstream antisense region of transcription start sites (TSSs) promotes early termination in PAS RNAs, which is subject to the nuclear RNA exosome-mediated degradation. In contrast, the high density of U1 snRNP recognition sites in the sense direction suppresses promoter-proximal cleavage events in the sense direction and thus allows productive elongation of Pol II to licence robust coding gene transcription [42] (Figure. 1(c)).

In summary, the fate of PAS RNAs is closely monitored and tightly regulated by multiple mechanisms. The precise control of PAS RNAs homoeostasis, combined with unique nuclear retention expression pattern, implies that PAS RNAs, that were previously thought to be transcriptional by-products, may function as molecular entities in gene regulation. Indeed, antisense transcripts and their corresponding biological functions have previously been reviewed in elsewhere [31,43]. Prior studies have used various approaches to knock down PAS RNAs (*e.g*. using small interfering RNAs [siRNAs], antisense oligonucleotides [ASOs], locked nucleic acids [LNAs]), or edit their genomic sequence. However, these approaches can be confounded by the effects of such perturbations on DNA regulatory elements or transcriptional machinery. Therefore, while previous studies have reported effects of PAS RNAs, whether the PAS RNAs per se are functional is still controversial. In the next section, I will review the alternative approaches we used in our recent study to unambiguously demonstrate that PAS RNAs act as functional RNA molecules to licence ligand-induced transcription activation [44].

## PAS RNAs as functional molecules

The observation that PAS RNA/mRNA pairs exhibit positive correlated expression changes in response to differentiation or stress stimulation triggers poses an intriguing question as to whether PAS RNA could directly regulate cognate coding gene transcription [9,44]. A classic strategy to determine such a function is to perturb its gene expression (loss of function) or overexpression (gain of function), followed by the cognate coding gene expression analysis. However, the unique location pattern of PAS RNA (that is, PAS RNA usually locates at promoter region where it is transcribed) makes it challenging to manipulate PAS RNA expression without affecting other biological processes, such as transcription activation or promoter activity. For example, an siRNA based PAS RNA knockdown approach may exert its effect by potential genomic silencing at the promoter region [45–48]. An ASO based PAS RNA knockdown approach may achieve coding gene repression by perturbation of PAS RNA transcription rather than by depletion of PAS RNA per se [49,50]. CRISPR-Cas9 (Clustered Regularly Interspaced Short Palindromic Repeats) based editing or repression strategy has a direct impact on changing DNA sequence or promoter activity, which restricts its application for investigation of PAS RNA function. A conventional overexpression approach does not apply for studying the function of most, if not all, of PAS RNAs that function *in cis*. Hence, there remains a need for new methodology that is able to specifically manipulate PAS RNAs and thus determine the putative function of the promoter derived antisense transcripts.

The recent discovery of CRISPR-Cas13 system, a novel type of RNA guided RNA targeting enzymes, adds to the arsenal of versatile RNA manipulation applications [51]. One of the most straightforward applications for CRISPR-Cas13 is stable and robust targeted RNA degradation in mammalian cells. We tested the possibility by using oestrogen (E_2_)-induced transcription activation system. Oestrogen Receptor α (ERα) is a ligand-dependent sex steroid-regulated transcription factor that mediates most of the biological effects of E_2_, acting primarily at the level of gene transcription [52]. E_2_-bound ERα on enhancers causes a global increase in eRNA transcription on the ~1,300 enhancers adjacent to E_2_-upregulated coding genes that result in increased strength of specific enhancer-promoter looping [53]. E_2_ stimulation results in rapid *trans*-recruitment of an ERα-dependent MegaDalton sized protein complex, referred to as the MegaTrans complex [54]. GRO-seq approaches reveal that E_2_ regulates a large transcriptional program based on Pol II promoter-proximal pause release where a large cohort of PAS RNAs (837 PAS RNAs) and their corresponding coding gene transcripts are robustly induced [26,44]. By using gRNAs to target LwaCas13a (Cas13a from *Leptotrichia wadei*) to selectively degrade PAS RNAs, we have provided direct evidence for the functional importance of induced PAS RNAs in the transcriptional activation of cognate coding genes by E_2_ in breast cancer cells. Furthermore, to prove PAS RNAs as functional molecules in regulating coding gene expression, we specifically overexpressed several control RNAs and PAS RNAs at selected promoters by using CRISPR-dCas9 (catalytically dead Cas9)-mediated PAS RNA tethering approach. Our data demonstrates that only specific PAS RNA overexpression, but not control RNA overexpression at selected promoters is sufficient to promote coding gene transcription. Therefore, to the best of our knowledge, our endeavour to employ CRISPR-Cas13a- based RNA knockdown strategy and CRISPR-dCas9- based PAS RNA tethering strategy, for the first time, unequivocally proves that PAS RNAs are functional entities in gene regulation.

## Role of PAS RNA in Pol II promoter-proximal pause release regulation

Transcription, transmitting genetic information from DNA to RNA, plays a fundamental role in nearly all the physiopathological processes and its regulation is precisely regulated by multistep mechanisms [55–57]. Among them is the critical role of transcription elongation, which is characterized by Pol II promoter-proximal pause release. This transcription process, which plays a critical regulatory role for genes that respond to various developmental and environmental cues [58], has been increasingly recognized in the literature. Transcription regulation has recently been recognized to be strongly associated with dynamic change of post-translational modification of histone proteins and non-coding RNAs [59,60], but how a stimulus dependent epigenetic event, as well as PAS RNAs that specifically locate at promoter regions, contributes to Pol II promoter-proximal pause release remains poorly understood. The evidence presented in our study suggests that E_2_-induced PAS RNAs, acting as functional molecules, regulate Pol II promoter-proximal pause release in a large subset of genes based on their actions to ensure erasure of H3K9me3 (histone H3 tri-methylation at Lysine 9) promoter mark by stabilization of H3K9me3 demethylases KDM4B and KDM4C binding, via a sharing of similar stem-loop cluster RNA structure of PAS RNAs, at promoters. This results in a dismissal of the previously-overlooked H3K9me3 reader proteins HP1α and HP1β assembly that occurs at these euchromatic promoters. We provide evidence that the promoter-bound HP1α and HP1β are capable of stabilizing 7SK small nuclear ribonucleoprotein (snRNP) complex and NELFA, leading to promoter-proximal Pol II pausing, and gene repression. Together, these results indicate that HP1 (including both HP1α and HP1β) stabilizes 7SK snRNP complex and NELFA assembly on paused promoters, and that E_2_- induced PAS RNA ensures dismissal of HP1-7SK-NELFA assembly for robust coding gene transcription.

## PAS RNA structure in gene regulation

**Figure 2.**
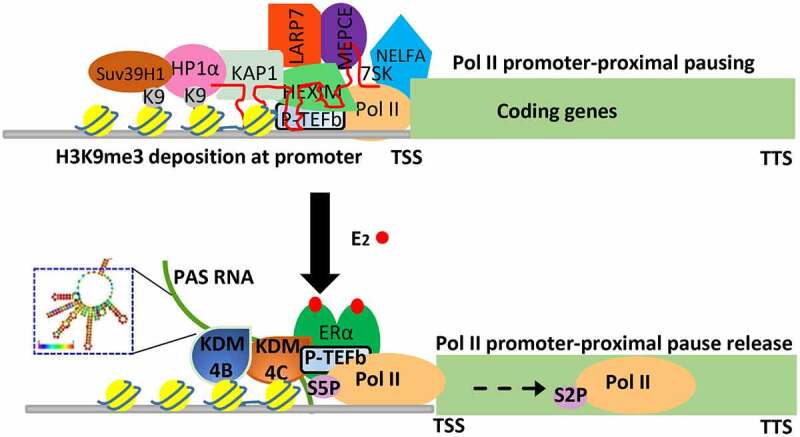
Functional importance of PAS RNA in ligand-induced transcription activation

As with proteins, RNA molecules form complex higher-order structures that are indispensable regulators of many biological processes [61]. For instance, ribosomal RNA depends on scaffolding RNA as a key assembly platform to fold into well-defined secondary and tertiary structures for regulating protein translation [62]. The lncRNA *Xist* enables appropriate X chromosome inactivation (XCI) through its distinct well-ordered structural domains [63]. Recent technological advance in transcriptome-wide interrogation of RNA structure and new computational modelling approach has dramatically transformed our understanding of RNA linking its sophisticated structures to its diverse functions [64]. However, the inherent technic limitation of current methods, prevents them from genome-wide structural measurement of most unstable transcripts, including PAS RNAs. Our study presents evidence showing that *ABAT* PAS RNA, which is an E_2_- induced promoter antisense transcript, is critical for regulation of Pol II promoter-proximal pause release and the cognate coding gene transcription activation via a stable and compact stem-loop structure. Indeed, while the primary nucleotide sequences of various E_2_-induced PAS RNAs on promoters exhibiting Pol II promoter-proximal pause release-dependent activation vary widely, here we provide evidence that they share a similar stem-loop cluster, and that this RNA structure is responsible for the recruitment of the H3K9me3 demethylases KDM4B and KDM4C, which serves as the underlying mechanism by which PAS RNAs serve to regulate Pol II promoter-proximal pause release events based on recruitment of H3K9me3 demethylases that release HP1α recruited to promoters (Figure. 2).

## Concluding remarks and future perspectives

Rapid ever-evolving genome-wide nascent RNA sequencing technologies have revealed prevalent expression landscape of PAS RNAs across all the investigated genome so far. Despite the pervasiveness of PAS RNAs expression, a full understanding of the mechanisms by which they might regulate gene expression has remained rather enigmatic. Indeed, although accumulating data generated in the last decades points towards diverse functions of PAS RNAs in a myriad of biological processes, direct evidence to prove PAS RNAs themselves, rather than the act of transcription in the upstream antisense direction of promoter regions, in such processes remains inadequate.

In this Review, I have described two of the most powerful approaches that enable efficient genome-wide detection of PAS RNAs. I have summarized the most recent understanding of PAS RNA biogenesis and regulation, and have recapitulated our recent work to prove PAS RNAs as functional entities in gene regulation, thus supporting our novel mechanistic insights into PAS RNAs- mediated gene regulation. Nevertheless, it should be noted that although the example of PAS RNA mechanism described above suggests a specific function for PAS RNAs in epigenetic regulation, there remains an uncertainty of the precise roles and mechanisms of most of PAS RNAs. A recent study reports a fascinating model whereby the negatively charged nascent RNAs- mediated electrostatic interaction with positively charged proteins regulates Pol II transcription via a dynamic complex coacervation model [65], which can also be applied to PAS RNAs. Therefore, comprehensive functional studies of PAS RNAs will be necessary in forthcoming work within various contexts and will be instrumental in establishing how general the function of PAS RNAs is as critical regulators of transcriptional activation.

Ascribed to most, if not all, of PAS RNAs functioning in *cis*, it is currently technically difficult to query PAS RNAs in a systemic level. Further efforts are needed to achieve genome-wide perturbation of PAS RNAs. Intriguingly, some studies have also reported an opposite gene repression effect of antisense transcription on coding gene expression. Hence, a subset of PAS RNAs, in specific cellular contexts, may have a direct role in gene repression. Therefore, it is pertinent to dissect whether PAS RNAs have both transcriptional activation and repression function and what determines their functional specification. Additionally, it is necessary to combine CRISPR-Cas13- based PAS RNA knockdown strategy and CRISPR-dCas9- based PAS RNA tethering strategy presented above to distinguish between effects caused by PAS RNA per se and the act of transcription in the upstream antisense direction of promoter region. It is noteworthy that both models underlying PAS RNA functions are not mutually exclusive and may co-exist in some contexts, in terms of the vast number of PAS RNAs in a variety of genomes. It is certainly exciting to investigate the ability of specific structural features or nucleotide sequence elements in PAS RNA biology. Given the increasingly recognized importance of RNA structure in RNA function, it is particularly cogent to test and identify whether there is a ‘structure code’ for PAS RNAs allowing them to establish a dynamic and specific interaction with proteins, RNAs and other macromolecules, thus licencing the formation of ribonucleoprotein complexes essential for key regulatory functions of PAS RNAs. In parallel, attention should be paid to potential modifications of PAS RNAs, which will greatly deepen our mechanistic insights into PAS RNAs. With continually evolving technological advances in nascent RNA sequencing, RNA secondary structure probing and modification approaches, the functional repertoire of PAS RNAs will expand and revolutionize our fundamental understanding of the broader role of these previously neglected transcripts.
